# Analysis of risk factors for early elevated intraocular pressure after vitrectomy in patients with diabetes

**DOI:** 10.1097/MD.0000000000049654

**Published:** 2026-07-17

**Authors:** Yan Dai, Anlang Dai, Runjia Lei

**Affiliations:** a Department of Ophthalmology, Lanzhou Petrochemical General Hospital, Lanzhou City, Gansu Province, China; b Department of Anesthesiology, Lanzhou Petrochemical General Hospital, Lanzhou City, Gansu Province, China; c Department of Ophthalmology, Xi’an No.1 Hospital (The First Affiliated Hospital of Northwest University), Xi’an City, Shaanxi Province, China.

**Keywords:** diabetic retinopathy, postoperative elevated intraocular pressure, risk factors, risk prediction model, vitrectomy

## Abstract

This study aimed to investigate the incidence and independent risk factors of early elevated intraocular pressure (IOP) after vitrectomy in patients with diabetes and establish a clinical risk prediction model. A retrospective case study was conducted, including 130 patients (130 eyes) who underwent vitrectomy for diabetic retinopathy from June 2022 to June 2024. Patients were divided into 2 groups based on whether early postoperative elevated IOP occurred within 1 week after surgery (defined as IOP ≥25 mm Hg or an increase ≥10 mm Hg compared with preoperative values). Demographic data, systemic conditions, preoperative ocular characteristics, and intraoperative parameters were collected. Univariate and multivariate logistic regression analyses were performed to identify independent risk factors. A risk scoring system was constructed accordingly, and its predictive performance was evaluated using the receiver operating characteristic curve. The incidence of early postoperative elevated IOP was 30.0% (39/130). Multivariate logistic regression analysis showed that higher preoperative glycated hemoglobin (odds ratio [OR] = 1.57, 95% confidence interval [CI]: 1.17–2.11), combined cataract surgery (OR = 3.47, 95% CI: 1.19–10.15), use of intraocular tamponade (expansile gas: OR = 4.96; silicone oil: OR = 10.15), and longer operative time (OR = 1.23) were independent risk factors for early postoperative elevated IOP (all *P* < .05). The clinical risk scoring system (total score 0–9) based on these factors had an area under the receiver operating characteristic curve of 0.86 (95% CI: 0.79–0.93). With a cutoff value of ≥4 points, the sensitivity was 82.10% and the specificity was 78.00%. According to the score, patients were classified into low-, medium-, and high-risk groups, with actual incidences of elevated IOP of 8.1%, 28.3%, and 63.2%, respectively. The incidence of early postoperative elevated IOP after vitrectomy is high in patients with diabetes. High preoperative glycated hemoglobin, combined cataract surgery, use of silicone oil or gas tamponade, and prolonged operative time are independent risk factors. The clinical risk scoring system established in this study demonstrates good predictive ability and may facilitate early identification of high-risk patients for targeted intervention.

## 1. Introduction

Diabetic retinopathy (DR) is one of the leading causes of blindness worldwide, and with the continuous increase in the prevalence of diabetes, the burden of vision impairment caused by DR continues to rise annually.^[1,2]^ Pars plana vitrectomy (PPV) plays a crucial role in the management of complications such as proliferative diabetic retinopathy (PDR), vitreous hemorrhage, tractional retinal detachment, and diabetic macular edema, and has become an important means to improve visual function.^[3,4]^ However, a series of postoperative complications may occur after PPV, among which early elevated intraocular pressure (early postoperative elevated IOP) is one of the most common and clinically significant conditions.^[5]^ Previous studies have shown that the overall incidence of elevated IOP after vitrectomy can reach 20% to 50%. Due to more pronounced inflammatory reactions, higher surgical complexity, and poor glycemic control, diabetic patients may face an even higher risk of postoperative IOP elevation.^[6]^ Early postoperative IOP elevation not only affects visual recovery but may also lead to optic nerve damage, retinal perfusion impairment, and even secondary glaucoma, resulting in irreversible deterioration of final visual function. Therefore, early identification of risk factors and timely intervention are of great clinical importance.^[7,8]^

Although studies on postoperative IOP changes after PPV in diabetic patients have been reported, the systematic evaluation of associated risk factors remains insufficient, particularly the lack of quantitative tools that can predict risk during the preoperative and intraoperative stages. Existing studies have suggested that poor glycemic control, combined cataract surgery, longer operative time, and different types of intraocular tamponade may all influence postoperative IOP.^[9]^ However, the conclusions of current evidence are not entirely consistent, and most studies have not established clinically applicable prediction models, making preoperative risk assessment still largely dependent on clinical experience. Given the pronounced ocular inflammatory response, complex surgical procedures, and significant postoperative physiological changes in diabetic patients, the establishment of a risk prediction system based on real-world data is particularly necessary.

Based on the above background, this study retrospectively included DR patients who underwent PPV in our hospital from June 2022 to June 2024. By systematically collecting patients’ systemic conditions, preoperative ocular characteristics, and intraoperative parameters, the study analyzed the incidence and related risk factors of early postoperative elevated IOP within 1 week after surgery. Multivariate logistic regression analysis was further used to identify independent risk factors, and on this basis, a clinical risk scoring system was constructed. The aim was to provide clinicians with a concise, effective, and practical tool for identifying high-risk patients during the preoperative period and early postoperative stage, thereby guiding individualized intervention strategies and reducing visual impairment caused by postoperative IOP elevation. The implementation of this study not only contributes to a deeper understanding of the mechanisms underlying postoperative IOP changes in diabetic patients undergoing vitrectomy but also provides important reference for optimizing perioperative management.

## 2. Materials and methods

### 2.1. Study methods

This study was approved by the Ethics Committee of Xi’an No.1 Hospital. This study was a retrospective case review including 130 patients (130 eyes) diagnosed with DR and undergoing PPV in our hospital from June 2022 to June 2024. Patients were grouped according to the occurrence of early elevated IOP within 1 week after surgery. Early elevated IOP was defined as IOP ≥ 25 mm Hg at any postoperative time point or an increase ≥10 mm Hg compared with preoperative baseline. According to these criteria, among the 130 eyes, 39 eyes (30.0%) were assigned to the early postoperative elevated IOP group and 91 eyes (70.0%) to the non-early postoperative elevated IOP group.

### 2.2. Inclusion and exclusion criteria

#### 2.2.1. Inclusion criteria

Clear diagnosis: age ≥ 18 years, with a confirmed diagnosis of diabetes by the endocrinology department or relevant specialty.

Surgical indication: vitrectomy required for DR, such as PDR or diabetic macular edema.

Surgical approach: standard 23G or 25G PPV performed in our hospital.

Normal baseline IOP: preoperative IOP stable within the range of 10 to 21 mm Hg without the use of IOP-lowering medications.

Complete data: complete preoperative clinical data and at least 1 week of postoperative follow-up with available early postoperative IOP records.

#### 2.2.2. Exclusion criteria

Nondiabetic ocular diseases: surgery primarily targeting nondiabetic ocular disorders (e.g., idiopathic epiretinal membrane, macular hole, nondiabetic retinal detachment).

History of glaucoma: preoperative diagnosis of glaucoma or ocular hypertension, or previous antiglaucoma surgery.

Other IOP-related diseases: presence of active uveitis, severe ocular trauma, lens-induced glaucoma, or other ocular conditions significantly affecting IOP.

Severe perioperative complications: severe intraoperative or postoperative complications within 1 week requiring reoperation (e.g., fulminant endophthalmitis, complex retinal detachment).

Incomplete data: missing clinical information or early postoperative loss to follow-up, making effective evaluation impossible.

### 2.3. Data collection

To minimize selection bias, consecutive patients who met the predefined inclusion and exclusion criteria during the study period were enrolled. Standardized electronic medical records and surgical reports were used for data extraction. Two investigators independently reviewed clinical records, and discrepancies were resolved by consensus to reduce information bias. Nevertheless, because of the retrospective design, unmeasured confounding factors and residual bias cannot be completely excluded. We collected 4 categories of data from patients who met the inclusion and exclusion criteria, as detailed below.

#### 2.3.1. Demographic and systemic information

To evaluate the impact of systemic health status on postoperative IOP, we collected patients’ basic demographic data and systemic health indicators. These included age and sex; height and weight measured at admission, from which body mass index was calculated. Additionally, diabetes-related variables were recorded, including disease duration (years) and the most recent preoperative glycated hemoglobin (HbA1c) level to assess long-term glycemic control. Common systemic comorbidities were also documented, specifically whether patients had been diagnosed with hypertension, coronary heart disease, or renal insufficiency by relevant specialties.

#### 2.3.2. Preoperative ocular characteristics

The assessment of preoperative ocular status aimed to eliminate confounding factors and identify potential risks. The collected data included preoperative stable IOP measurements and axial length obtained using an ocular biometer. Based on preoperative fundus photography and fluorescein angiography, 2 senior ophthalmologists classified DR into nonproliferative (NPDR) and proliferative (PDR) stages. Previous ocular interventions were also recorded, including any history of vitrectomy or panretinal photocoagulation. Lens status was confirmed by slit-lamp examination and categorized as clear lens/pseudophakia or cataract.

#### 2.3.3. Intraoperative procedures and surgical parameters

All procedures were performed by 2 senior vitreoretinal surgeons with more than 10 years of surgical experience using standardized surgical protocols. The indications for tamponade selection and combined cataract surgery were based on routine departmental practice. No significant differences in surgical techniques were identified between surgeons.

In addition, all patients received a standardized perioperative medication regimen, including topical corticosteroids and antibiotics after surgery. Prophylactic IOP-lowering medications were not routinely administered unless postoperative IOP elevation occurred according to predefined criteria.

Intraoperative data collection focused on analyzing the direct surgical effects on IOP. The following variables were extracted from surgical records: gauge of the vitrectomy system (23G or 25G) and total operative time (minutes). Combined cataract surgery, including phacoemulsification and intraocular lens (IOL) implantation, was specifically recorded. For core vitreoretinal procedures, surgical videos or operative notes were reviewed to determine whether extensive membrane peeling was performed, and the extent of intraoperative laser photocoagulation was graded (local/moderate vs extensive). The type of intraocular tamponade used at the end of surgery was documented in detail as balanced salt solution/air, expansile gas (e.g., perfluoropropane [C3F8], sulfur hexafluoride [SF6]), or silicone oil. Any recorded intraoperative complications (such as iatrogenic breaks or significant bleeding) were also included in the analysis.

#### 2.3.4. Postoperative follow-up and outcome data

The primary focus of postoperative follow-up data was monitoring and defining the study outcome. Daily IOP measurements within 1 week after surgery were collected for all patients. Based on the predefined criteria – IOP ≥ 25 mm Hg at any postoperative time point or an increase ≥ 10 mm Hg from the preoperative baseline – patients were categorized into the early postoperative elevated IOP group and the non-elevated IOP group for subsequent comparison and risk factor analysis.

### 2.4. Statistical analysis

Statistical analysis was performed using SPSS 26.0 software. Continuous variables were expressed as mean ± standard deviation and compared between groups using independent-sample *t*-tests. Categorical variables were presented as counts (percentages) and compared using the χ^2^ test. Variables with *P* < .1 in the univariate analysis were included in the multivariate logistic regression analysis to identify independent risk factors for early postoperative elevated IOP. A clinical risk scoring system was constructed based on the regression results, and its predictive performance was assessed using the receiver operating characteristic curve. A *P* value < .05 was considered statistically significant.

## 3. Results

### 3.1. Comparison of baseline characteristics and preoperative ocular features

There were no statistically significant differences between the 2 groups in terms of age, sex, body mass index, duration of diabetes, prevalence of hypertension, coronary heart disease, or renal insufficiency, as well as preoperative IOP, axial length, DR stage, history of prior surgery, or history of laser treatment (*P* > .05). However, the preoperative HbA1c level in the early postoperative elevated IOP group was significantly higher than that in the non-elevated IOP group (8.9% ± 1.6% vs 7.9% ± 1.4%, *P* < .001). In addition, there was a significant difference in the distribution of lens status between the 2 groups, with the early postoperative elevated IOP group showing a higher proportion of cataract (74.4% vs 56.0%, *P* = .044; see Table 1).

**Table 1:** Comparison of baseline characteristics and preoperative ocular findings between the early elevated IOP group and non-elevated IOP group.

Variables	Total (n = 130)	Early elevated IOP (n = 39)	Non-elevated IOP (n = 91)	Statistic	*P* value
Demographics and systemic conditions					
Age (yr, mean ± SD)	58.5 ± 9.2	59.8 ± 8.7	58.0 ± 9.4	*t* = 1.05	.296
Sex (male), n (%)	72 (55.4%)	20 (51.3%)	52 (57.1%)	χ^2^ = 0.41	.522
Body mass index (kg/m^2^, mean ± SD)	25.1 ± 3.3	25.5 ± 3.5	24.9 ± 3.2	*t* = 0.98	.329
Duration of diabetes (yr, mean ± SD)	12.4 ± 5.1	13.5 ± 4.8	11.9 ± 5.2	*t* = 1.68	.095
Preoperative HbA1c (%, mean ± SD)	8.2 ± 1.5	8.9 ± 1.6	7.9 ± 1.4	*t* = 3.72	<.001
Hypertension, n (%)	85 (65.4%)	28 (71.8%)	57 (62.6%)	χ^2^ = 1.07	.301
Coronary artery disease, n (%)	22 (16.9%)	8 (20.5%)	14 (15.4%)	χ^2^ = 0.52	.471
Renal insufficiency, n (%)	18 (13.8%)	7 (17.9%)	11 (12.1%)	χ^2^ = 0.82	.365
Preoperative ocular characteristics					
Preoperative IOP (mm Hg, mean ± SD)	15.3 ± 2.8	15.1 ± 3.0	15.4 ± 2.7	*t* = –0.58	.565
Axial length (mm, mean ± SD)	23.2 ± 0.9	23.1 ± 0.8	23.3 ± 0.9	*t* = –1.21	.228
DR staging, n (%)				χ^2^ = 1.25	.264
Nonproliferative DR (NPDR)	35 (26.9%)	8 (20.5%)	27 (29.7%)		
Proliferative DR (PDR)	95 (73.1%)	31 (79.5%)	64 (70.3%)		
Previous vitrectomy, n (%)	15 (11.5%)	6 (15.4%)	9 (9.9%)	χ^2^ = 0.83	.362
Prior panretinal photocoagulation, n (%)	80 (61.5%)	26 (66.7%)	54 (59.3%)	χ^2^ = 0.64	.424
Lens status, n (%)				χ^2^ = 4.07	.044
Clear lens/pseudophakia	50 (38.5%)	10 (25.6%)	40 (44.0%)		
Cataract	80 (61.5%)	29 (74.4%)	51 (56.0%)		

HbA1c = glycated hemoglobin, IOP = intraocular pressure, NPDR = nonproliferative diabetic retinopathy, PDR = proliferative diabetic retinopathy, SD = standard deviation.

### 3.2. Comparison of intraoperative conditions

Comparison of intraoperative characteristics showed that the proportion of patients undergoing combined cataract phacoemulsification with IOL implantation was significantly higher in the early postoperative elevated IOP group than in the non-elevated IOP group (84.6% vs 62.6%, *P* = .009). The distribution of intraocular tamponade types also differed markedly between the 2 groups (*P* < .001), with the early postoperative elevated IOP group showing a much higher proportion of silicone oil tamponade (51.3% vs 16.5%). In addition, the operative time was significantly longer in the early postoperative elevated IOP group (92 ± 25 min vs 82 ± 20 min, *P* = .016). There were no significant differences between the 2 groups in terms of vitrectomy system gauge, extensive membrane peeling, extent of laser photocoagulation, or incidence of intraoperative complications (see Table 2).

**Table 2:** Comparison of intraoperative characteristics between the early elevated IOP group and non-elevated IOP group.

Variables	Total (n = 130)	Early elevated IOP (n = 39)	Non-elevated IOP (n = 91)	Statistic	*P* value
Surgical characteristics					
Surgical gauge system, n (%)				χ^2^ = 0.35	.554
23-gauge	75 (57.7%)	24 (61.5%)	51 (56.0%)		
25-gauge	55 (42.3%)	15 (38.5%)	40 (44.0%)		
Combined phacoemulsification + IOL implantation, n (%)	90 (69.2%)	33 (84.6%)	57 (62.6%)	χ^2^ = 6.78	.009
Extensive membrane peeling, n (%)	95 (73.1%)	32 (82.1%)	63 (69.2%)	χ^2^ = 2.38	.123
Extent of intraoperative laser photocoagulation, n (%)				χ^2^ = 1.40	.237
Local/moderate	60 (46.2%)	15 (38.5%)	45 (49.5%)		
Extensive	70 (53.8%)	24 (61.5%)	46 (50.5%)		
Tamponade materials, n (%)				χ^2^ = 26.15	<.001
BSS/air	50 (38.5%)	5 (12.8%)	45 (49.5%)		
Expansile gas	45 (34.6%)	14 (35.9%)	31 (34.1%)		
Silicone oil	35 (26.9%)	20 (51.3%)	15 (16.5%)		
Duration of surgery (min, mean ± SD)	85 ± 22	92 ± 25	82 ± 20	*t* = 2.45	.016
Intraoperative complications, n (%)	18 (13.8%)	7 (17.9%)	11 (12.1%)	χ^2^ = 0.82	.365

BSS = balanced salt solution, IOL = intraocular lens, IOP = intraocular pressure.

### 3.3. Multivariate logistic regression analysis of early postoperative elevated IOP

The results of multivariate logistic regression analysis (Table 3) showed that higher preoperative HbA1c levels, combined cataract phacoemulsification with IOL implantation, use of intraocular tamponade, and longer operative time were independent risk factors for early postoperative elevated IOP after vitrectomy in diabetic patients. Specifically, for every 1% increase in preoperative HbA1c, the risk of early postoperative elevated IOP increased by 57% (odds ratio [OR] = 1.57, 95% confidence interval [CI]: 1.17–2.11, *P* = .003). Combined cataract surgery during PPV significantly increased the risk 3.47-fold (OR = 3.47, 95% CI: 1.19–10.15, *P* = .023). Compared with balanced salt solution or air tamponade, the use of expansile gas and silicone oil was associated with substantially higher risks, increasing the risk 4.96-fold (OR = 4.96, 95% CI: 1.46–16.82, *P* = .010) and 10.15-fold (OR = 10.15, 95% CI: 3.20–32.21, *P* < .001), respectively. In addition, for every 10-minute increase in operative time, the risk of early postoperative elevated IOP increased by 23% (OR = 1.23, 95% CI: 1.01–1.48, *P* = .037).

**Table 3:** Multivariable logistic regression analysis of risk factors for early elevated intraocular pressure.

Variable	β	SE	Wald χ^2^	*P* Value	OR	95% CI for OR
Preoperative HbA1c (per 1% increase)	0.452	0.152	8.84	.003	1.57	1.17–2.11
Lens status (Ref: clear lens/pseudophakia)						
Cataract	0.891	0.512	3.03	.082	2.44	0.89–6.65
Combined phacoemulsification + IOL (yes)	1.245	0.548	5.16	0.023	3.47	1.19–10.15
Tamponade material (Ref: BSS/air)						
Expansile gas	1.602	0.623	6.61	.01	4.96	1.46–16.82
Silicone oil	2.318	0.589	15.49	<.001	10.15	3.20–32.21
Duration of surgery (per 10-min increase)	0.204	0.098	4.33	.037	1.23	1.01–1.48
Duration of diabetes (per 5-year increase)	0.211	0.182	1.34	.247	1.23	0.86–1.76
Extensive membrane peeling (yes)	0.567	0.522	1.18	.277	1.76	0.63–4.91
Constant	−12.245	2.876	18.13	<.001		

BSS = balanced salt solution, CI = confidence interval, HbA1c = glycated hemoglobin, OR = odds ratio, SE = standard deviation.

### 3.4. Construction of a clinical risk scoring system for early postoperative elevated IOP based on multivariate analysis

To facilitate rapid clinical risk assessment, a clinical risk scoring system for early postoperative elevated IOP was developed based on the independent risk factors identified in the multivariate logistic regression analysis (Table 4). The system incorporates 4 core indicators: preoperative HbA1c level, type of intraocular tamponade, whether combined cataract surgery was performed, and operative time. Each indicator was assigned a score according to its relative risk magnitude, with silicone oil tamponade receiving the highest score (3 points), followed by combined cataract surgery (2 points) and expansile gas tamponade (2 points). The total risk score for each patient was calculated as the sum of all item scores, with a theoretical range of 0 to 9 points.

**Table 4:** Clinical risk scoring system for predicting early elevated intraocular pressure after vitrectomy.

Risk factor	Category	Assigned score
Preoperative HbA1c (%)	<8.0	0
	8.0–8.9	1
	≥9.0	2
Tamponade material	Balanced salt solution/air	0
	Expansile gas	2
	Silicone oil	3
Combined phacoemulsification and IOL implantation	No	0
	Yes	2
Duration of surgery (min)	<80	0
	80–99	1
	≥100	2
Total possible risk score	–	0–9 points

BSS = balanced salt solution, HbA1c = glycated hemoglobin, IOL = intraocular lens.

### 3.5. Predictive performance and clinical stratification validation of the risk scoring system

**Figure 1.**
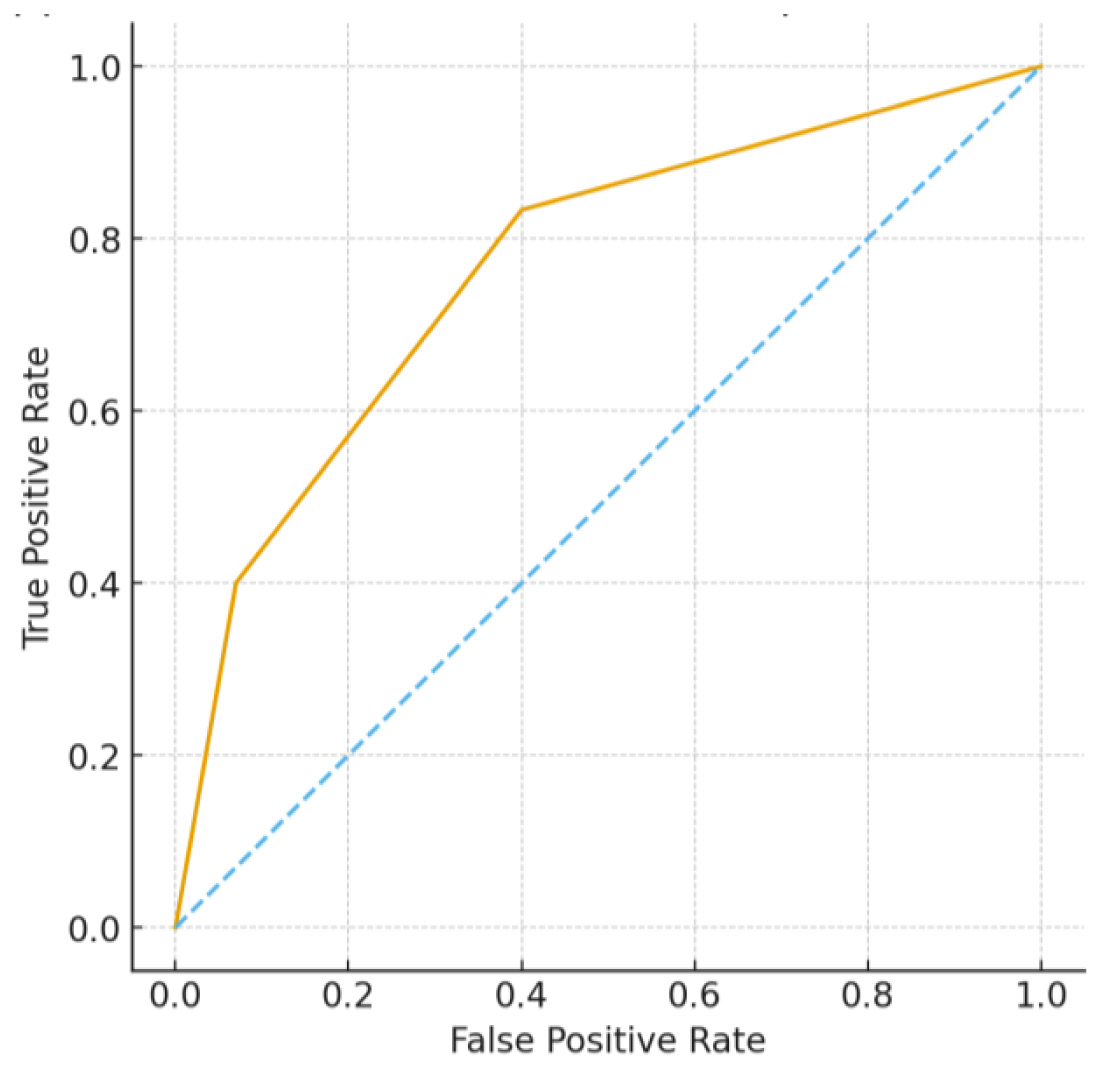
Receiver operating characteristic (ROC) curve of the clinical risk scoring model for predicting early elevated intraocular pressure after vitrectomy. ROC = receiver operating characteristic.

To evaluate the predictive value of the scoring system, a receiver operating characteristic curve was generated (Fig. 1). The area under the curve was 0.86 (95% CI: 0.79–0.93), indicating good predictive and discriminative ability for early postoperative elevated IOP. The optimal diagnostic cutoff value, determined by the Youden index, was ≥4 points. At this threshold, the sensitivity of the model was 82.10% and the specificity was 78.00% (Table 5).

**Table 5:** Predictive performance of the clinical risk score for early elevated intraocular pressure.

Analysis item	Value
Area under the ROC curve (AUC)	0.86 (95% CI: 0.79–0.93)
Optimal cutoff value (Youden index)	≥ 4 points
Sensitivity at cutoff ≥ 4	82.10%
Specificity at cutoff ≥ 4	78.00%
Positive predictive value	61.50%
Negative predictive value	91.00%
Youden index	0.6
Risk stratification	Observed incidence of early elevated IOP
Low-risk group (0–2 points)	8.1% (5/62)
Intermediate-risk group (3–5 points)	28.3% (13/46)
High-risk group (6–9 points)	63.2% (12/19)

AUC = area under the curve, IOP = intraocular pressure, ROC = receiver operating characteristic.

Furthermore, patients were stratified into 3 risk levels according to their total scores: low-risk group (0–2 points), moderate-risk group (3–5 points), and high-risk group (6–9 points). The actual observed incidence of early postoperative elevated IOP showed a clear gradient across these categories: 8.1% (5/62) in the low-risk group, 28.3% (13/46) in the moderate-risk group, and 63.2% (12/19) in the high-risk group. These findings confirm the effectiveness and clinical utility of the scoring system for risk stratification.

## 4. Discussion

This study systematically evaluated the incidence and associated risk factors of early elevated IOP after vitrectomy in patients with diabetes and constructed a clinical risk prediction model based on multivariate analysis. The results showed that the incidence of early postoperative elevated IOP was 30.0%, which is relatively high and indicates the need for increased clinical attention to this complication in diabetic patients.^[10]^ Elevated preoperative HbA1c levels, combined cataract surgery, the use of expansile gas or silicone oil tamponade, and prolonged operative time were identified as independent risk factors for early postoperative elevated IOP. Moreover, the risk scoring system established in this study demonstrated high predictive accuracy for early postoperative elevated IOP, with an area under the curve of 0.86, further supporting its potential utility in preoperative assessment and postoperative management.

A higher preoperative HbA1c level was one of the most important systemic risk factors identified in this study.^[11]^ HbA1c reflects long-term glycemic control, and its elevation also indicates diabetes-associated inflammatory responses, microvascular impairment, and reduced tissue healing capacity.^[12]^ During surgery, the levels of inflammatory mediators such as vascular endothelial growth factor, interleukin-6, and tumor necrosis factor-alpha are typically higher in diabetic eyes, and poor glycemic control may further promote the release of inflammatory factors, disrupt aqueous humor dynamics, and ultimately increase trabecular meshwork resistance, leading to elevated IOP.^[13,14]^ In addition, patients with elevated HbA1c often exhibit more severe retinal ischemia and fragile neovascularization, which increases intraoperative difficulty and indirectly elevates the risk of postoperative IOP elevation.^[15]^ These findings suggest that optimizing glycemic control before surgery is not only essential for stabilizing retinal disease but may also serve as an important measure to reduce the risk of early postoperative elevated IOP.

Combined cataract surgery was also an important risk factor identified in this study. Vitrectomy combined with phacoemulsification is relatively common in diabetic patients, especially when lens opacity affects the surgical view. However, combined surgery may induce a more intense intraocular inflammatory response, leading to a temporary imbalance between aqueous humor production and outflow. Additionally, residual viscoelastic material, aqueous dynamics changes caused by corneal incisions, and other procedure-related factors may contribute to postoperative IOP elevation.^[16]^ This result supports the need for careful clinical evaluation of surgical necessity and postoperative risks. When feasible, staged surgical strategies may be considered, particularly for high-risk patients.

The impact of intraocular tamponade on postoperative IOP was especially pronounced. Gas tamponade may transiently increase intraocular volume due to its expansile properties, and volume fluctuations before complete absorption may also affect aqueous outflow pathways.^[17]^ In contrast, silicone oil tamponade exerts an even stronger effect on IOP. As a high-viscosity inert material, silicone oil may directly obstruct the angle structures, reduce aqueous outflow, or even migrate into the anterior chamber and cause mechanical resistance.^[18]^ In this study, the OR for silicone oil tamponade reached 10.15, indicating it as the strongest risk factor. This finding is consistent with previous reports, suggesting that silicone oil can induce elevated IOP by altering aqueous dynamics or forming emulsified droplets that block the trabecular meshwork. Therefore, for patients requiring silicone oil tamponade, meticulous IOP monitoring during the first postoperative week and timely administration of antiglaucoma medications are crucial.

Prolonged operative time was also confirmed to be closely associated with early postoperative elevated IOP. Operative time reflects, to some extent, surgical complexity and manipulation intensity. Complex membrane peeling, repeated hemostasis, and extensive laser photocoagulation may heighten tissue irritation and inflammatory responses, thereby reducing aqueous outflow.^[19,20]^ Additionally, extended procedures may increase the accumulation of cellular debris or biological particles within the aqueous circulation, further overloading the trabecular meshwork. This finding suggests that improving surgical efficiency, minimizing unnecessary maneuvers, and optimizing operative steps may help reduce the risk of elevated IOP.^[21]^

The risk scoring system constructed based on these key factors performed well in distinguishing patients with varying risk levels. It provides not only an objective basis for preoperative assessment but also supports targeted postoperative follow-up. In this study, the incidence of elevated IOP exceeded 60% in the high-risk group but was only 8.1% in the low-risk group, indicating that the scoring system can effectively guide individualized management strategies, including preoperative risk counseling, selection of tamponade materials during surgery, and intensified postoperative IOP monitoring and early intervention.

Despite its clinical value, this study has several limitations. First, as a single-center retrospective study with a relatively small sample size, this study may be subject to selection bias and information bias inherent to retrospective data collection. Although consecutive case inclusion and standardized data extraction procedures were adopted to minimize these biases, residual confounding from unmeasured variables cannot be entirely excluded. Therefore, caution is warranted when interpreting the estimated effect sizes. In addition, this study focused primarily on early IOP changes within 1 week after surgery and did not include long-term IOP control or subsequent glaucoma outcomes. Importantly, the present scoring system should be regarded as a preliminary predictive tool derived from a single-center retrospective cohort. Although it demonstrated good discrimination in the current dataset, external validation and calibration using larger prospective multicenter cohorts are essential before widespread clinical implementation. The use of standardized surgical protocols and perioperative management strategies may partially reduce variability related to operator-dependent factors.

In conclusion, this study identified multiple independent risk factors for early postoperative elevated IOP after vitrectomy in diabetic patients and successfully established a risk scoring system with good predictive performance. Although promising, this scoring system should currently be considered an exploratory clinical aid rather than a definitive predictive instrument, pending further external validation. For patients with elevated HbA1c levels, requiring combined cataract surgery, treated with expansile gas or silicone oil tamponade, or undergoing longer surgeries, more intensive perioperative management strategies should be implemented to reduce adverse effects associated with postoperative IOP elevation and improve long-term visual outcomes.

## Author contributions

**Conceptualization:** Yan Dai, Anlang Dai, Runjia Lei.

**Data curation:** Yan Dai, Anlang Dai, Runjia Lei.

**Formal analysis:** Yan Dai, Anlang Dai, Runjia Lei.

**Funding acquisition:** Runjia Lei.

**Writing – original draft:** Yan Dai, Anlang Dai, Runjia Lei.

**Writing – review & editing:** Yan Dai, Anlang Dai, Runjia Lei.

## References

[1] Yau JW Rogers SL Kawasaki R. Global prevalence and major risk factors of diabetic retinopathy. Diabetes Care. 2012;35:556–64. 22301125 10.2337/dc11-1909 PMC3322721

[2] Leasher JL Bourne RR Flaxman SR. Global estimates on the number of people blind or visually impaired by diabetic retinopathy: a meta-analysis from 1990 to 2010. Diabetes Care. 2016;39:1643–9. 27555623 10.2337/dc15-2171

[3] De Maria M Panchal B Coassin M. Update on indications for diabetic vitrectomy and management of complications. Ann Eye Sci. 2018;3:51–51.

[4] Smiddy WE Feuer W Irvine WD Flynn HW Jr Blankenship GW. Vitrectomy for complications of proliferative diabetic retinopathy. Functional outcomes. Ophthalmology. 1995;102:1688–95. 9098263 10.1016/s0161-6420(95)30808-1

[5] Desai UR Alhalel AA Schiffman RM Campen TJ Sundar G Muhich A. Intraocular pressure elevation after simple pars plana vitrectomy. Ophthalmology. 1997;104:781–6. 9160023 10.1016/s0161-6420(97)30233-4

[6] Sharma YR Pruthi A Azad RV Kumar A Mannan R. Impact of early rise of intraocular pressure on visual outcome following diabetic vitrectomy. Indian J Ophthalmol. 2011;59:37–40. 21157070 10.4103/0301-4738.73724 PMC3032240

[7] Weinberg RS Peyman GA Huamonte FU. Elevation of intraocular pressure after pars plana vitrectomy. Albrecht von Graefes Archiv fur klinische und experimentelle Ophthalmologie. 1976;200:157–61. 1086606 10.1007/BF00414365

[8] Pournaras CJ Rungger-Brändle E Riva CE Hardarson SH Stefansson E. Regulation of retinal blood flow in health and disease. Prog Retin Eye Res. 2008;27:284–330. 18448380 10.1016/j.preteyeres.2008.02.002

[9] Fan W Zhang C Ge L. Prediction model for elevated intraocular pressure risk after silicone oil filling based on clinical features. Front Med (Lausanne). 2024;10:1340198. 38264037 10.3389/fmed.2023.1340198 PMC10803451

[10] Eslami Y Mohammadi M Fakhraie G Zarei R Moghimi S. Ahmed glaucoma valve implantation with tube insertion through the ciliary sulcus in pseudophakic/aphakic eyes. J Glaucoma. 2014;23:115–8. 22828010 10.1097/IJG.0b013e318265bc0b

[11] Jin P Peng J Zou H. A five-year prospective study of diabetic retinopathy progression in Chinese type 2 diabetes patients with “well-controlled” blood glucose. PLoS One. 2015;10:e0123449. 25849536 10.1371/journal.pone.0123449 PMC4388440

[12] Alswaina N. Association between HbA1c levels and the severity of diabetic retinopathy. Cureus. 2024;16:e76395. 39867069 10.7759/cureus.76395 PMC11762433

[13] Liu J Yu M Yan J. The association between glycated hemoglobin and intraocular inflammatory factors in patients with proliferative diabetic retinopathy. Int J Retina Vitreous. 2025;11:115. 41126323 10.1186/s40942-025-00732-y PMC12542134

[14] Wakabayashi Y Usui Y Okunuki Y. Intraocular VEGF level as a risk factor for postoperative complications after vitrectomy for proliferative diabetic retinopathy. Invest Ophthalmol Vis Sci. 2012;53:6403–10. 22899753 10.1167/iovs.12-10367

[15] Liang X Zhang Y Li YP Huang WR Wang JX Li X. Frequency and risk factors for neovascular glaucoma after vitrectomy in eyes with diabetic retinopathy: an observational study. Diabetes Ther. 2019;10:1801–9. 31321746 10.1007/s13300-019-0644-0 PMC6778559

[16] Gershoni A Barayev E Jbara D. Postoperative complications of combined phacoemulsification and pars plana vitrectomy in diabetic retinopathy patients. Front Med (Lausanne). 2022;9:978346. 36250076 10.3389/fmed.2022.978346 PMC9561423

[17] Dan H Wang D Huang Z. Comparison of the effectiveness of vitrectomy with silicone oil or perfluoropropane tamponade for myopic foveoschisis with foveal detachment. Front Med (Lausanne). 2025;12:1602386. 41064507 10.3389/fmed.2025.1602386 PMC12500636

[18] Ge L Su N Fan W Yuan S. Risk factors and management of intraocular pressure elevation after vitrectomy combined with silicone oil tamponade. Int J Gen Med. 2024;17:447–56. 38333017 10.2147/IJGM.S446617 PMC10849908

[19] Motoda S Shiraki N Ishihara T. Predictors of postoperative bleeding after vitrectomy for vitreous hemorrhage in patients with diabetic retinopathy. J Diabetes Investig. 2018;9:940–5. 10.1111/jdi.12791 PMC6031499 29265703

[20] Tolentino FI Cajita VN Gancayco T Skates S. Vitreous hemorrhage after closed vitrectomy for proliferative diabetic retinopathy. Ophthalmology. 1989;96:1495–500. 2587044 10.1016/s0161-6420(89)32700-x

[21] Han DP Lewis H Lambrou FH Jr Mieler WF Hartz A. Mechanisms of intraocular pressure elevation after pars plana vitrectomy. Ophthalmology. 1989;96:1357–62. 2780005 10.1016/s0161-6420(89)32715-1

